# Chest radiography in children aged 2–59 months enrolled in the Innovative Treatments in Pneumonia (ITIP) project in Lilongwe Malawi: a secondary analysis

**DOI:** 10.1186/s12887-021-03091-3

**Published:** 2022-01-10

**Authors:** Tisungane Mvalo, Eric D. McCollum, Elizabeth Fitzgerald, Portia Kamthunzi, Robert H. Schmicker, Susanne May, Melda Phiri, Claightone Chirombo, Ajib Phiri, Amy Sarah Ginsburg

**Affiliations:** 1 Lilongwe Medical Relief Fund Trust, University of North Carolina Project, Lilongwe, Malawi; 2 Department of Pediatrics, School of Medicine, University of North Carolina at Chapel Hill, Chapel Hill, North Carolina USA; 3 Global Program in Respiratory Sciences, Eudowood Division of Pediatric Respiratory Sciences, Department of Pediatrics, School of Medicine, Johns Hopkins University, Baltimore, MD USA; 4 Department of International Health, Bloomberg School of Public Health, Johns Hopkins University, Baltimore, MD USA; 5 Institute for Global Health and Infectious Diseases, University of North Carolina at Chapel Hill, Chapel Hill, North Carolina USA; 6 University of Washington Clinical Trial Center, Seattle, Washington USA; 7 Department of Pediatrics and Child Health, College of Medicine, University of Malawi, Lilongwe Campus, Lilongwe, Malawi

**Keywords:** Chest radiography, Childhood pneumonia

## Abstract

**Background:**

Pneumonia is the leading infectious cause of death in children aged under 5 years in low- and middle-income countries (LMICs). World Health Organization (WHO) pneumonia diagnosis guidelines rely on non-specific clinical features. We explore chest radiography (CXR) findings among select children in the Innovative Treatments in Pneumonia (ITIP) project in Malawi in relation to clinical outcomes.

**Methods:**

When clinically indicated, CXRs were obtained from ITIP-enrolled children aged 2 to 59 months with community-acquired pneumonia hospitalized with treatment failure or relapse. ITIP1 (fast-breathing pneumonia) and ITIP2 (chest-indrawing pneumonia) trials enrolled children with non-severe pneumonia while ITIP3 enrolled children excluded from ITIP1 and ITIP2 with severe pneumonia and/or selected comorbidities. A panel of trained pediatricians classified the CXRs using the standardized WHO CXR research methodology. We analyzed the relationship between CXR classifications, enrollee characteristics, and outcomes.

**Results:**

Between March 2016 and June 2018, of 114 CXRs obtained, 83 met analysis criteria with 62.7% (52/83) classified as having significant pathology per WHO standardized interpretation. ITIP3 (92.3%; 12/13) children had a higher proportion of CXRs with significant pathology compared to ITIP1 (57.1%, 12/21) and ITIP2 (57.1%, 28/49) (*p*-value = 0.008). The predominant pathological CXR reading was “other infiltrates only” in ITIP1 (83.3%, 10/12) and ITIP2 (71.4%, 20/28), while in ITIP3 it was “primary endpoint pneumonia”(66.7%, 8/12,; *p*-value = 0.008). The percent of CXRs with significant pathology among children clinically cured (60.6%, 40/66) vs those not clinically cured (70.6%, 12/17) at Day 14 was not significantly different (*p*-value = 0.58).

**Conclusions:**

In this secondary analysis we observed that ITIP3 children with severe pneumonia and/or selected comorbidities had a higher frequency of CXRs with significant pathology, although these radiographic findings had limited relationship to Day 14 outcomes. The proportion of CXRs with “primary endpoint pneumonia” was low. These findings add to existing data that additional diagnostics and prognostics are important for improving the care of children with pneumonia in LMICs.

**Trial registration:**

ITIP1, ITIP2, and ITIP3 were registered with ClinicalTrials.gov (NCT02760420, NCT02678195, and NCT02960919, respectively).

**Supplementary Information:**

The online version contains supplementary material available at 10.1186/s12887-021-03091-3.

## Introduction

Pneumonia is the leading infectious cause of death in children under the age of 5 years in low- and middle-income countries (LMICs) [1–4]. Ongoing morbidity and mortality persist despite the introduction of conjugate vaccines for *Streptococcus pneumoniae* and *Haemophilus influenzae* type b (Hib), major bacterial causes of childhood pneumonia.

World Health Organization (WHO) Integrated Management of Childhood Illness (IMCI) guidelines used to diagnose pneumonia in resource-constrained settings rely on non-specific clinical features including cough, difficulty breathing, fast breathing-for-age and chest indrawing [5]. When imaging is clinically indicated in children with suspected pneumonia, chest radiography (CXR) has long been considered the reference standard [6, 7]. In 2001, the WHO vaccine trials group developed a standardized research method for vaccine trials and epidemiological studies to interpret and define CXR changes in children likely attributable to bacterial pneumonia [6, 7].

CXR-confirmed pneumonia based on the WHO CXR methodology (WHO CXR-confirmed pneumonia) has been shown to correlate with laboratory features of infection or the isolation of pneumonia-causing pathogens such as *S. pneumoniae* in blood or respiratory samples [3, 8–10]. Compared to WHO IMCI-defined pneumonia, children with WHO CXR-confirmed pneumonia, are reported to have higher treatment failure rates, longer hospital stays, and higher morbidity and mortality in LMICs [9, 11–15]. Previous studies have demonstrated that no single clinical feature is adequate to predict WHO CXR-confirmed pneumonia [16–18]. However, with reports that children with pneumonia and abnormal CXRs have poorer outcomes, further data from sub-Saharan Africa that describes the indications for CXR imaging and the predictive value of CXR findings for pediatric outcomes appears prudent.

Patterns of WHO CXR-confirmed pneumonia may be shifting after the introduction of pneumococcal and Hib conjugate vaccines and with respiratory viruses becoming increasingly predominant [19, 20]*.* Few studies to date have examined whether CXR findings are associated with outcomes of children diagnosed with pneumonia per WHO IMCI guidelines in Africa post-introduction of pneumococcal and Hib conjugate vaccines. To address these gaps, we conducted a post-hoc secondary analysis to describe the clinical indications for CXR imaging and explore CXR findings among children 2 to 59 months of age with community-acquired pneumonia in the Innovative Treatments in Pneumonia (ITIP) project at Kamuzu Central Hospital (KCH) in Lilongwe, Malawi.

## Methods

### Setting and study population

The pediatric ward of KCH, a tertiary hospital in Lilongwe, has a 299-bed capacity that hospitalizes up to 25,000 children annually. The KCH radiology department performs approximately 9000 CXRs annually, and CXRs are requested by treating clinicians, largely for children with pneumonia either not responding to antibiotic treatment or deteriorating clinically. The KCH radiologist may be consulted for interpretation of CXRs when required, but CXRs are typically interpreted by the clinician providing care.

The ITIP project consisted of three studies: ITIP1, a double-blind randomized controlled clinical trial evaluating 3 days of amoxicillin dispersible tablets (DT) versus placebo DT (intervention) for non-severe fast-breathing pneumonia [21]; ITIP2, a double-blind randomized controlled clinical trial evaluating 5 versus 3 days (intervention) of amoxicillin DT for non-severe chest-indrawing pneumonia [22]; and ITIP3, a prospective observational study of clinical outcomes among children with pneumonia who were excluded from ITIP1 and ITIP2 due to severe pneumonia and/or comorbidities such as HIV exposure or infection, severe malaria, severe acute malnutrition, and/or anemia with hemoglobin < 8 g/Dl [23]. The ITIP3 study population included children with both severe and non-severe pneumonia. Children presenting to the study sites with a history of cough and/or difficulty breathing were screened for eligibility to enroll in one of the three ITIP studies. Children enrolled in the ITIP1 or ITIP2 clinical trials were managed by ITIP study clinicians.

Those ITIP1 and ITIP2 children who demonstrated treatment failure while receiving oral study drug - amoxicillin DT or placebo DT - at Day 4 (ITIP1) or Day 6 (ITIP2) assessment or who had clinical relapse of pneumonia after Day 4 (ITIP1) or Day 6 (ITIP2) and by Day 14 follow-up visits, were hospitalized and received intravenous antibiotic treatment with benzyl penicillin and gentamicin (first-line intravenous antibiotic regimen). The pneumonia diagnosis at the time of initiating intravenous antibiotics was either fast-breathing pneumonia, chest-indrawing pneumonia, danger sign pneumonia, or CXR-confirmed pneumonia (Table 1). In the children requiring hospitalization for pneumonia relapse, the clinical pneumonia diagnosis at the time of hospitalization could either be the same or different from the clinical diagnosis at time of enrollment. ITIP1 and ITIP2 children with treatment failure to benzyl penicillin and gentamicin were switched to ceftriaxone (second-line intravenous antibiotic regimen) by ITIP study clinicians. Children enrolled in the ITIP3 observational study were managed at the discretion of non-study KCH clinicians. Typically, ITIP3 children with danger sign pneumonia were hospitalized and placed on intravenous benzyl penicillin and gentamicin. Some ITIP3 children were subsequently changed to second-line intravenous antibiotic treatment with ceftriaxone due to an assessment of poor response or deterioration while on intravenous benzyl penicillin and gentamicin. ITIP3 children with HIV infection or exposure were treated as outpatients if they did not have danger signs.

**Table 1:** Study definitions

Terminology	Definition
World Health Organization (WHO) Integrated Management of Childhood Illness (IMCI) general danger signs	Lethargy or unconsciousness, convulsions, vomiting everything, or inability to drink or breastfeed
Respiratory danger signs	Grunting, nasal flaring, head nodding, severe chest indrawing, or hypoxemia (pulse oximetry saturation < 90%)
Fast-breathing pneumonia	History of cough < 14 days or difficult breathing with fast breathing-for-age (> 50 breaths/minute in children 2 to < 12 months of age, > 40 breaths/minute in children > 12 months of age) in the absence of chest indrawing and WHO IMCI general and respiratory danger signs
Chest-indrawing pneumonia	History of cough < 14 days or difficult breathing with chest indrawing in the absence of WHO IMCI general and respiratory danger signs
Danger sign pneumonia	History of cough < 14 days or difficult breathing and the presence of WHO IMCI general and/or respiratory danger signs
Chest radiograph (CXR)-confirmed pneumonia	History of cough < 14 days or difficulty breathing with CXR features of pneumonia per the assessment of the clinician interpreting the CXR in the absence of fast breathing-for-age, chest indrawing, and WHO general and respiratory danger signs
First-line intravenous antibiotic treatment failure	Persistence or presence of new WHO general or respiratory danger signs after at least 2 days of receiving intravenous benzyl penicillin and gentamicin
Second-line intravenous antibiotic treatment failure	Persistence or presence of new WHO general or respiratory danger signs after at least 5 days of receiving intravenous ceftriaxone

### Data collection

Per ITIP study standard operating procedures, anteroposterior CXRs were obtained from ITIP1 and ITIP2 children who were hospitalized and treated with second-line intravenous antibiotics due to treatment failure to first-line intravenous antibiotics, who had persistent pneumonia or relapse of pneumonia at Day 14 (study exit visit), or who had treatment failure due to isolated fever while on oral study drugs. As ITIP3 was an observational study, anteroposterior CXRs for ITIP3 children were obtained at the clinical discretion of the non-study KCH clinician. Digital CXR images were acquired from a Philips Medical Systems 9890 00002031 Optimus 50 x-ray machine and printed into analogue format for interpretation by CXR readers. All CXRs were independently interpreted in printed format by three interpreters masked to each other’s interpretations. The three interpreters, EF, PK and TM, were trained in the WHO standardized interpretation of pediatric CXRs by a member of the WHO Chest Radiography in Epidemiological Studies working group (EDM), and all three passed standardized testing prior to performing the interpretations for this study [6, 7, 24]. TM was the third interpreter and arbitrator in cases where there were discordant CXR interpretations between the first (EF) and second (PK) interpreters.

WHO standardized interpretation of pediatric CXRs (Table 2) was undertaken to optimize comparisons with existing epidemiological and clinical studies [24]. Thus, CXR readings were divided into four categories: “primary endpoint pneumonia only”; “other infiltrates only”; “primary endpoint pneumonia and other infiltrates”; and “no significant pathology” (no pneumonia, no infiltrates or effusion) [6, 7, 25]. The first three interpretation categories were considered to be CXR interpretations with significant pathology. During data analysis, “primary end point pneumonia only” and “primary endpoint point pneumonia and other infiltrates” were combined into one category namely, “primary endpoint pneumonia”.

**Table 2:** World Health Organization standardized interpretation of pediatric anteroposterior chest radiographs in pneumonia epidemiological studies (adapted from Mahomed et al., 2017)

Film Quality	Definition
Uninterpretable	Features of the image are not interpretable with respect to presence or absence of consolidation or pleural effusion without additional images.
Suboptimal	Features allow interpretation of consolidation and pleural effusion, but not of other infiltrates or findings.
Adequate	Features allow confident interpretation of consolidation and pleural effusion as well as other infiltrates.
**Classification of findings**	
Significant pathology	Refers specifically to the presence of consolidation, infiltrates or effusion.
Endpoint consolidation	A dense or confluent opacity that occupies a portion or whole of a lobe or the entire lung that may or may not contain air bronchogram.
Other (non-endpoint) infiltrates	Linear and patchy opacities (interstitial infiltrate) in a lacy pattern, featuring Peribronchial thickening and multiple areas of atelectasis; it also includes minor patchy infiltrates that are not of sufficient magnitude to constitute endpoint consolidation, and small areas of atelectasis that in children may be difficult to distinguish from consolidation.
Pleural effusion	Presence of fluid in the lateral pleural space between the lung and chest wall that is spatially associated with a pulmonary parenchymal infiltrate (including other infiltrate) or has obliterated enough of the hemithorax to obscure any infiltrate; in most cases, this will be seen at the costo-phrenic angle or as a layer of fluid adjacent to the lateral chest wall; this does not include fluid seen in the horizontal or oblique fissures.
**Conclusions**	
Primary endpoint pneumonia (PEP).	The presence of consolidation or pleural effusion, (as defined above)
Other infiltrate	The presence of other (non-consolidation) infiltrates as defined above in the absence of a pleural effusion.
No consolidation/infiltrate/effusion	Absence of consolidation, other infiltrates or pleural effusion.

Data were retrieved from the ITIP project databases and safety reports, and included age, sex, presence or absence of WHO general and/or respiratory danger signs, mid-upper arm circumference, malaria rapid diagnostic test result, HIV rapid diagnostic test result, intravenous antibiotic regimen, duration of hospital stay, and Day 14 outcome of pneumonia illness.

**Fig. 1:**
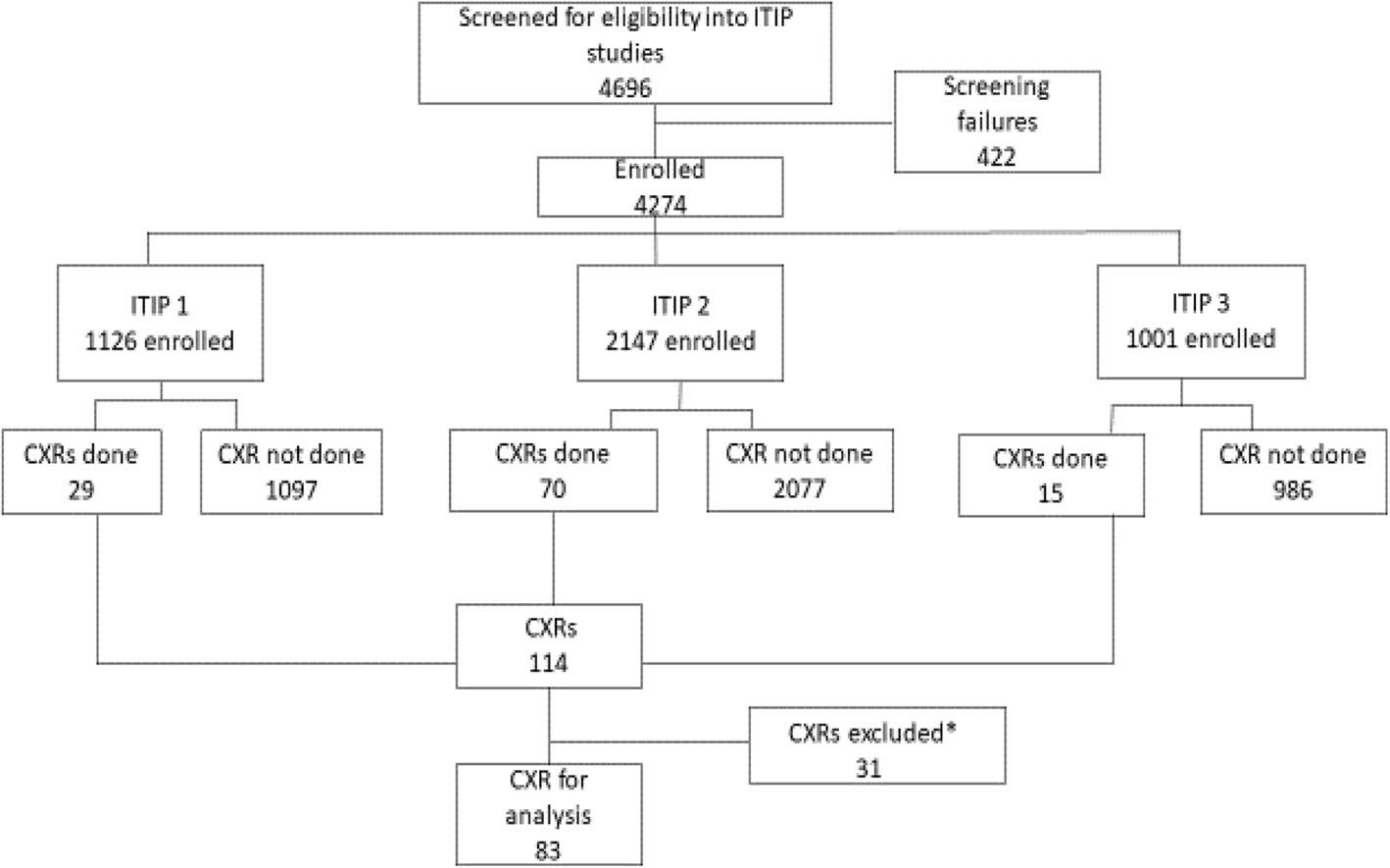
Consort diagram of children who had CXR across the ITIP studies. Of the 31 CXRs excluded from this analysis, 13 were from children not hospitalized, 7 were uninterpretable CXRs, 4 had CXR reading documentation inconsistencies and 7 were duplicate CXRs

Conducted from March 2016 to April 2019, the three ITIP studies enrolled 5127 (ITIP1, 1126; ITIP2, 3000; ITIP3, 1001) children. However, this secondary CXR analysis included children enrolled between March 2016 and June 2018 only as this was the period when all WHO CXR methodology-trained pediatricians were available at the study site for the reading of the printed CXRs. From March 2016 and June 2018, the three ITIP studies enrolled 4274 children ies and 114 CXRs were obtained (Fig. 1). This analysis includes all CXRs obtained for enrolled ITIP1 and ITIP3 children, and for ITIP2 children enrolled through June 2018.

### Data analysis

Clinical and safety data from the three ITIP studies were merged with the CXR findings and indications for performing CXRs. Children were excluded from this analysis if they were not hospitalized, if the CXR was uninterpretable, or if there were inconsistencies in CXR documentation. Child and CXR characteristics were stratified by ITIP study and compared by chi-squared or analysis of variance tests, depending on variable type. Pneumonia type, treatment failure, and Day 14 outcomes were stratified by CXR classification and compared by Fisher’s exact tests. Concordance of CXR findings was calculated as the percentage of full matches of the CXR reading categories between the first two reviewers (EF and PK). Extracted data was tabulated on excel (Additional file 1.cxr data) and analyzed per data dictionary (Additional file 2. data dictionary). Analysis was conducted in R v3.5.1 (Vienna, Austria) [26].

## Results

Of the 83 enrolled ITIP children whose CXRs were included in this analysis, 24.1% (21/83) were from ITIP1, 60.9% (49/83) were from ITIP2, and 14.9% (13/83) were from ITIP3 (Table 3). The majority of children in all three studies were aged 2 to 11 months. Other baseline characteristics were similar for children across the three studies with the exception of a trend for a lower mean mid-upper arm circumference in ITIP3 compared to ITIP1 and ITIP2.

**Table 3:** Baseline characteristics of children at time of enrollment into the Innovative Treatments in Pneumonia (ITIP) project at who had a chest radiograph for ITIP1 (fast-breathing pneumonia), ITIP2 (chest-indrawing pneumonia), and ITIP3 (pneumonia with comorbidities and/ or danger signs) studies

Child characteristic	ITIP1 ***N*** = 21	ITIP2 ***N*** = 49	ITIP3 ***N*** = 13	Total ***N*** = 83	***p***-value
Sex (female), n (%)	8 (38.1)	18 (36.7)	3 (23.1)	29 (34.9)	0.62
Age (months), n (%)					0.02
2–11	11 (52.4)	33 (67.3)	8 (61.5)	52 (62.7)	
12–35	7 (33.3)	14 (28.6)	5 (38.5)	26 (31.3)	
36–59	3 (14.3)	2 (4.1)	0 (0)	5 (6.0)	
Median (IQR)	11 (6, 25)	7 (3, 13)	10 (2, 21)	8 (3, 15.5)	
Mid-upper arm circumference (mm), n (%)					< 0.001
< 115	0 (0.0)	0 (0.0)	1 (7.7)	1 (1.2)	
115–135	2 (9.5)	17 (34.7)	5 (38.5)	24 (28.9)	
> 135	19 (90.5)	32 (65.3)	7 (53.8)	58 (69.9)	
Positive malaria rapid diagnostic test, n (%)	1 (4.8)	2 (4.1)	3 (23.1)	6 (7.2)	0.06
Diarrhea present, n (%)	0 (0.0)	4 (8.2)	1 (7.7)	5 (6.0)	0.41
Fever (≥ 38 ͦ C), n (%)	7 (33.3)	10 (20.4)	3 (23.1)	20 (24.1)	0.55

NOTE: *p*-values for sex, malaria, diarrhea, and fever were obtained from chi-squared tests. *p*-values for age and mid-upper arm circumference were from analysis of variance

CXRs demonstrating significant pathology were identified in 62.7% (52/83) of children across the three studies (Table 4). ITIP3 children demonstrated a higher proportion of significant pathology on CXRs (92.3%, 12/13) in comparison to similar proportions across ITIP1 (57.1%, 12/21) and ITIP2 (57.1%, 28/49) children (*p*-value = 0.008). Among those CXRs demonstrating significant pathology, “other infiltrates only” (65.4%, 34/52) was the most frequent interpretation, followed by “primary endpoint pneumonia” (34.6%, 18/52). “Other infiltrates only” was the most frequent interpretation in ITIP1 (83.3%, 10/12) and ITIP2 (71.4%, 20/28), while “primary endpoint pneumonia” was the most frequent interpretation in ITIP3 (66.744%, 8/12). These differences noted in CXR interpretations across the three ITIP studies were statistically significant (*p*-value = 0.008). Of note, no CXR demonstrated a pleural effusion and 21.7% (18/83) had a reading of “primary endpoint pneumonia.”

**Table 4:** Chest radiograph (CXR) findings, clinical diagnoses at time of CXR, and CXR indications for ITIP1 (fast-breathing pneumonia), ITIP2 (chest-indrawing pneumonia), and ITIP3 (pneumonia with comorbidities and/ or danger signs) studies

n (%)	ITIP1 ***N*** = 21	ITIP2 ***N*** = 49	ITIP3 ***N*** = 13	Total ***N*** = 83	***p***-value
CXR findings					0.008
CXR with no significant pathology	9 (42.9)	21 (42.9)	1 (7.7)	31 (37.3)	
CXR with significant pathology	12 (57.1)	28 (57.1)	12 (92.3)	52 (62.7)	
Primary endpoint pneumonia^a^	2 (16.7)	8 (28.6)	8 (66.7)	18 (34.6)	
Other infiltrates only	10 (83.3)	20 (71.4)	4 (33.3)	34 (65.4)	
Pleural effusion	0 (0)	0 (0)	0 (0)	0 (0)	
Clinical diagnoses at time of CXR					0.006
Fast-breathing pneumonia	3 (14.3)	7 (14.3)	0 (0.0)	10 (12.0)	
Chest-indrawing pneumonia	5 (23.8)	22 (44.9)	4 (30.8)	31 (37.4)	
Danger sign pneumonia	3 (14.3)	15 (30.6)	7 (53.8)	25 (30.1)	
Other^b^	10 (47.6)	5 (10.2)	2 (15.4)	17 (20.5)	
CXR indications					0.001
First-line intravenous antibiotic treatment failure	4 (19.0)	17 (34.7)	3 (23.1)	24 (28.9)	
Isolated fever	9 (42.9)	1 (2.0)	1 (7.7)	11 (13.3)	
Treatment failure/relapse at Day 14	5 (23.8)	24 (49.0)	6 (46.2)	35 (42.2)	
Other^c^	3 (14.3)	7 (14.3)	3 (23.1)	13 (15.7)	

*p*-values for CXR findings and CXR indications were obtained from Fisher’s exact tests; *p*-value for clinical diagnoses was obtained from chi-square test

^a^This category consists of CXR readings of primary endpoint pneumonia with or without other infiltrates

^b^Other clinical diagnoses included 10 CXR-confirmed pneumonia cases, 6 isolated fever cases, and 1 severe acute malnutrition case in which pulmonary tuberculosis was being investigated

^c^Other indications for CXR included 10 cases with an unclear or undocumented reason for ordering a CXR by requesting clinician, 1 case investigating possible foreign body aspiration, 1 case of isolated fever, and 1 case of suspected pulmonary tuberculosis

When comparing clinical findings to CXR findings, danger sign pneumonia was present in 50% (9/18) of the CXR interpretations of “primary endpoint pneumonia” while danger sign and chest-indrawing pneumonias were present in 26.5% (9/34) of the interpretations of “other infiltrates only” (Table 5). “Primary endpoint pneumonia” was not observed in any children with fast-breathing pneumonia. Variations in CXR interpretations by WHO IMCI clinical pneumonia diagnoses or durations of hospital stay were not statistically significant.

**Table 5:** Chest radiograph (CXR) findings in relation to clinical findings across ITIP studies

Clinical variable, n (%)	CXR findings			Total N (%)	***p***-value
	Primary endpoint pneumonia ***N*** = 18	Other infiltrates only ***N*** = 34	No significant pathology ***N*** = 31	N = 83	
Hospital duration^a^					
1–5 days	4 (22.2)	11 (32.4)	6 (19.4)	21 (25.3)	0.50
6–10 days	9 (50.0)	15 (44.1)	16 (51.6)	40 (48.2)	
11–15 days	3 (16.7)	7 (22.6)	6 (19.4)	16 (19.3)	
≥ 16 days	1 (5.6)	0 (0)	3 (9.7)	4 (4.8)	
Clinical pneumonia diagnosis^b^					
Fast-breathing pneumonia	0 (0)	5 (14.7)	5 (16.3)	10 (12.0)	0.08
Chest-indrawing pneumonia	5 (27.8)	9 (26.5)	17 (54.8)	31 (37.3)	
Danger sign pneumonia^c^	9 (50.0)	9 (26.5)	7 (22.6)	25 (30.1)	
Treatment failure on intravenous antibiotics					0.09
Treatment failure on first-line intravenous antibiotics	9 (50.0)	9 (26.5)	12 (38.7)	30 (36.1)	
Treatment failure on second-line intravenous antibiotics	5 (27.8)	0 (0)	1 (3.2)	6 (7.2)	
Day 14 assessment					
Cured at time of Day 14 visit	11 (61.1)	29 (85.3)	26 (83.9)	66 (79.5)	0.11
Not cured at time of Day 14 visit	7 (38.9)	5 (14.7)	5 (16.1)	17 (20.5)	

*p*-value for hospital duration was obtained via analysis of variance. Hospital duration variables were missing for 2 children. Values for pneumonia diagnosis, treatment failure on intravenous antibiotics, and Day 14 assessment were obtained from a Fisher’s exact test

^a^Hospital duration variables were missing for 2 children

^b^The total from the WHO pneumonia diagnosis equals 66; 17 children who had a CXR did not fall into this clinical diagnosis (10 had CXR-confirmed pneumonia, 6 had isolated fever, and 1 had severe acute malnutrition)

^c^Among children with danger sign pneumonia, 17 had severe chest indrawing, 11 nasal flaring, 5 head nodding, 2 grunting, and 2 hypoxemia

Treatment failure after first-line intravenous antibiotics was observed in 36.1% (30/83) of the ITIP children who had an interpretable CXR, 60% (18/30) of whom had a CXR demonstrating significant pathology. “Primary endpoint pneumonia” on CXR was noted in 30% (9/30) and “other infiltrates only” on CXR was present in 30% (9/30) of cases of treatment failure to first-line intravenous antibiotics. Treatment failure after second-line intravenous antibiotics occurred in 6 children. Among these children, 83% (5/6) had a CXR demonstrating significant pathology. “Primary endpoint pneumonia” was the CXR interpretation in all 5 of these children The proportion of CXRs demonstrating significant pathology among patients clinically cured at Day 14, compared to patients not clinically cured, was not significantly different (60.6% (40/66) vs 70.6% (12/17); *p*-value = 0.58). One ITIP3 child in this analysis died and the CXR showed “primary endpoint pneumonia.”

## Discussion

We found important variation in CXR interpretations among children enrolled in the three ITIP studies that was largely consistent with the child’s risk profile. CXRs demonstrating “primary endpoint pneumonia” or “other infiltrates only” were observed more frequently in the higher-risk ITIP3 children with severe pneumonia and/or comorbidities. On the other hand, children in ITIP1 and ITIP2 were by trial design low-risk, and we found their CXRs demonstrated either “no significant pathology” or “other infiltrates only” more frequently. CXRs with “primary endpoint pneumonia” were noted in 22% of all ITIP CXRs analyzed. Overall, the CXR patterns we observed may suggest that in the post-pneumococcal and Hib conjugate vaccine era, low-risk African children with WHO IMCI non-severe pneumonia who are failing antibiotic treatment are likely to have either nonbacterial lower respiratory infections or non-pulmonary illnesses altogether, while higher-risk children with severe pneumonia and/or comorbidities who are also failing antibiotics may have bacterial lower respiratory infections less responsive to primary antibiotic therapies.

CXR findings may help identify children with a higher or lower probability of bacterial or viral lower respiratory infection etiology. More specifically, CXRs with “other infiltrates only” may have a higher probability of viral etiology and CXRs with “primary endpoint pneumonia” may have a higher probability of bacterial etiology, while a combination of the two may suggest both viruses and bacteria as causative pathogens [3, 7, 8, 11]. The PERCH studies in children hospitalized with severe pneumonia in Africa and Asia reported 61.4% as having viral etiology [27]. Studies in South Africa reported the isolation of at least one virus in 87% of pneumonia cases [28]. Other studies have shown associations between higher-risk, severe cases of pneumonia with polymicrobial infection [19]. In our ITIP studies, the predominance of “other infiltrates only” (65%) on CXR implies viruses may be important pathogens in pneumonia cases in Malawi post-pneumococcal and Hib conjugate vaccine introduction. While we identified “other infiltrates only” frequently in our analysis, a study in India prior to the introduction of pneumococcal conjugate vaccine found “primary endpoint pneumonia” readings to be more frequent among CXRs from children with pneumonia [29]. In this analysis, we did not observe any statistically significant relationship between CXR findings and Day 14 clinical outcomes. However, point estimates suggest a higher proportion of children with ‘primary endpoint pneumonia’ may have remained ill on Day 14 than those without ‘primary endpoint pneumonia.’ Apart from antibiotic susceptibility of the pneumonia-causing pathogen, patient factors such as comorbidities, severity of illness on presentation, and inflammatory responses may affect the speed of recovery [30]. It is possible these may be effect modifiers on the relationship between CXR findings and outcomes. Given the relatively small sample size of this study, a larger study is needed to further explore this possibility.

Similar to other studies in LMICs that used standardized WHO research methodology to interpret CXRs, our analysis revealed nearly 40% of ITIP children with CXRs and first-line intravenous antibiotic failure were without significant CXR pathology. Furthermore, most children lacking CXR pathology were low-risk since they were classified as non-severe and lacked comorbidities [17, 31, 32]. It is notable that having a CXR without significant pathology does not exclude pneumonia in a child with respiratory signs. CXRs frequently miss alveolar consolidation identifiable on chest-computed tomography or the development of radiological abnormalities may lag the clinical presentation [18, 33, 34]. While chest computed tomography demonstrates high sensitivity and specificity for parenchymal lung disease, its routine use is infeasible in children, especially those in resource-constrained LMICs, given high ionizing radiation exposure and costs [35, 36]. Biomarkers like procalcitonin may offer an alternative to increase sensitivity for bacterial pneumonia or predicting those at risk of adverse outcomes [37, 38].

Among higher-risk children failing antibiotics, CXRs may be clinically indicated in part to exclude complications like pleural effusions [39]. It may be that we failed to identify any pleural effusions in the ITIP studies due to the limited number of CXRs collected or that pleural effusions were too small to visualize. All CXRs obtained in ITIP were supine anteroposterior images as this is the standard approach for imaging children at KCH. Supine CXR images can limit the detection of small pleural effusions, and while erect posterioanterior CXR images are preferred, they are not commonly obtained in young children due to challenges with compliance [40]. In addition, the absence of pleural effusions on CXR in our study may also be due to the changing epidemiology of pneumonia post-pneumococcal and Hib conjugate vaccine introduction [10]. A South African study evaluating children suspected of having pulmonary tuberculosis reported a higher proportion of pleural effusions using lung ultrasound (LUS) than CXR (15% versus 9%) [41]. Another study in Italy identified 28 more small-volume pleural effusions with LUS compared to CXR, among 47 children with pneumonia [42]. Our findings suggest clinicians may consider utilizing other imaging modalities like LUS rather than CXR to exclude pleural effusions.

Among children with suspected pneumonia confirmed by chest computed tomography, LUS has demonstrated a sensitivity and accuracy of 0.906 and 0.661, respectively in comparison to CXR which had a sensitivity and accuracy of 0.79 and 0.559, respectively [35]. Furthermore, LUS expert interpreters have achieved substantially higher interrater reliability compared to CXR expert interpreters [43]. Without ionizing radiation as a limitation, LUS may be useful to monitor treatment changes and possibly differentiate features of viral versus bacterial pneumonia [44–46]. LUS may also identify features of acute respiratory distress syndrome or lung changes noted in malaria and sepsis [47]. Early identification of children with acute respiratory distress or changes from malaria may improve their outcomes if appropriately triaged and managed. However, both CXR and LUS have their limitations. Aside from the difficulty in routinely capturing high-quality images in many resource-constrained settings, CXRs may have interpretation inaccuracies due to the reading of overlapping intrathoracic tissue and LUS may have challenges in identifying more centrally located lesions and lung abscesses [35, 48].

This study has several limitations. Our small sample size of hospitalized children receiving CXR examinations limits the generalizability of our results. In the ITIP studies, CXRs were ordered when clinically indicated rather than as a routine research procedure, which is reflective of actual pediatric clinical practice in LMICs. Given the current paucity of similar contemporary studies from LMICs, we believe the analysis of this relatively small sample adds hypothesis generating value in light of its rigor and real-world application. Another limitation to this project was the use of the WHO methodology for CXR interpretation. Although the WHO methodology is often applied to epidemiologic studies, the approach was originally designed to prioritize specificity and reproducibility for identifying children likely to have bacterial pneumonia participating in vaccine efficacy and effectiveness studies. For example, this approach does not take into account mediastinal or hilar lymphadenopathy that may suggest pulmonary tuberculosis, or other important findings like cardiomegaly or pneumothorax. This methodological gap limits our ability to comment on other findings that may explain the lack of improvement in children imaged with CXRs in ITIP. In the post-pneumococcal and Hib conjugate vaccine era, this suggests WHO-defined abnormal CXRs are more frequently associated with antibiotic failure [49].

## Conclusions

The lower frequency of CXRs with significant pathology among low-risk children failing oral antibiotic treatment suggests clinicians should consider more aggressive pursuit of alternative non-pneumonia diagnoses when antibiotic failure occurs. In our study, among higher-risk children, CXRs consistent with a bacterial lower respiratory infection did not predict Day 14 outcomes. The proportion of CXRs with primary endpoint pneumonia among children with a clinical diagnosis of pneumonia was low. The development and validation of alternative diagnostics, prognostics, and/or ancillary tests that can augment the yield and/or replace CXRs will be important for improving the care and outcomes of children with pneumonia in LMICs.

## Supplementary Information

**Additional file 1.** cxr data.

**Additional file 2.** Data dictionary.

## Data Availability

All data generated or analysed for this analysis are included in this published article and supplementary information files.

## Abbreviations

CXR: Chest radiography
DT: Dispersible tablets
Hib: *Haemophilus influenzae* type b
IMCI: Integrated Management of Childhood Illness
ITIP: Innovative Treatments in Pneumonia
KCH: Kamuzu Central Hospital
LMIC: Low- and middle-income countries
LUS: Lung ultrasound
WHO: World Health Organization.
